# Molecular Mechanisms of Hawthorn Extracts in Multiple Organs Disorders in Underlying of Diabetes: A Review

**DOI:** 10.1155/2022/2002768

**Published:** 2022-06-07

**Authors:** Izadpanah Gheitasi, Feryal Savari, Ghaidafeh Akbari, Jamshid Mohammadi, Ali Reza Fallahzadeh, Hossein Sadeghi

**Affiliations:** ^1^Medicinal Plants Research Center, Yasuj University of Medical Sciences, Yasuj, Iran; ^2^Department of Basic Sciences, Shoushtar Faculty of Medical Sciences, Shoushtar, Iran; ^3^Cellular and Molecular Research Center, Yasuj University of Medical Sciences, Yasuj, Iran

## Abstract

Diabetes mellitus (DM) is one of the most important metabolic disorders associated with chronic hyperglycemia and occurs when the body cannot manage insulin secretion, insulin action, or both. Autoimmune destruction of pancreatic beta cells and insulin resistance are the major pathophysiological factors of types 1 and 2 of DM, respectively. Prolonged hyperglycemia leads to multiple organs dysfunctions, including nephropathy, neuropathy, cardiomyopathy, gastropathy, and micro- and macrovascular disorders. The basis of the metabolic abnormalities in carbohydrate, fat, and protein in diabetes is insufficient action of insulin on various target tissues. Medicinal plants are rich sources of bioactive chemical compounds with therapeutic effects. The beneficial effects of leaves, fruits, and flowers extracts of *Crataegus oxyacantha,* commonly called hawthorn, belonging to the Rosaceae family, are widely used as hawthorn-derived medicines. Data in this review have been collected from the scientific articles published in databases such as Science Direct, Scopus, PubMed, Web of Science, and Scientific Information Database from 2000 to 2021. Based on this review, hawthorn extracts appear both therapeutic and protective effects against diabetic-related complications in various organs through molecular mechanisms, such as decreasing triglyceride, cholesterol, very low density lipoprotein and increasing the antioxidant activity of superoxide dismutase, catalase, glutathione peroxidase, total antioxidant capacity, decreasing malondialdehyde level, and attenuating tumor necrosis factor alpha, interleukin 6 and sirtuin 1/AMP-activated protein kinase (AMPK)/nuclear factor kappa B (NF-*κ*B) pathway and increasing the phosphorylation of glucose transporter 4, insulin receptor substrate 1, AKT and phosphoinositide 3-kinases, and attenuating blood sugar and regulation of insulin secretion, insulin resistance, and improvement of histopathological changes in pancreatic beta cells. Collectively, hawthorn can be considered as one new target for the research and development of innovative drugs for the prevention or treatment of DM and related problems.

## 1. Introduction

### 1.1. Diabetes Mellitus

Diabetes mellitus (DM) is one of the most common metabolic disorders marked by chronic hyperglycemia and occurs when the body is not able to properly manage insulin secretion, insulin function, or bot [1, 2]. Type 1 diabetes mellitus (T1DM) accounts for only 5–10% of those with diabetes [3]. Autoimmune destruction of pancreatic beta cells in T1DM leads to intensive insulin deficiency [4, 5]. Type 2 diabetes mellitus (T2DM) is a common type of DM in adults and accounts for approximately 90–95% of all diabetic individuals worldwide [3]. T2DM is manifested by insulin insensitivity because of insulin resistance, attenuating insulin production, and eventually pancreatic beta-cells dysfunction [6]. Insulin resistance, as the main pathophysiologic factor of T2DM, is manifested by disability of cells in glucose utilization [4, 7]. The basis of the metabolic abnormalities in carbohydrate, fat, and protein in diabetes is insufficient action of insulin in various target tissues [3]. Overweight, unhealthy dietary patterns, sedentary lifestyle, genetic background, aging, and stress are known to be implicated in the progression of T2DM [8].

Diabetic-related complications including hypertension, atherosclerosis, blindness, kidney failure, and stroke increase the mortality rate [9, 10]. DM is associated with typical diabetic Symptoms such as polydipsia, polyuria, polyphagia, and weight loss [11]. Furthermore, impairment of gastrointestinal function, numbness in lower extremities, and neuronal dysfunction may be accompanied with chronic hyperglycemia [12]. Hyperglycemia leads to the disruption of metabolic processes in multiple organs and results in retinopathy, nephropathy, and neuropathy [13, 14]. Oxidative stress and activation of proinflammatory reactions are other factors which contributed to the tissue damage following chronic hyperglycemia [15, 16].

### 1.2. Hawthorn

Medicinal plants as rich sources of bioactive chemical compounds have beneficial therapeutic effects [17]. *Crataegus oxyacantha,* commonly called hawthorn, belongs to the Rosaceae family. It is a fruit-bearing plant with green, yellow, orange, red, and black berries that grows mostly in Europe, Asia, North America, and Africa [18, 19]. The genus of *Crataegus* has more than 250 different species. The common name of hawthorn is currently used for all plant species of this genus [17, 19]. Hawthorn has gained growing attention due to its low toxicity and minimal unwanted side effects, which highlights its beneficial health effects and makes it easy to candidate as an alternative traditional medicine therapy. Leaves, fruits, and flowers extracts are widely used as the hawthorn-derived medicines [20, 21].

The leaves, flowers, and berries of hawthorn contain a variety of bioflavonoid-like complexes. Biflavonoids found in hawthorn plant include oligomeric procyanidins, vitexin, quercetin, and hyperoside. Other chemical constituents include vitamin C, saponins, tannins, cardiotonic amines (phenylethylamine, tyramine, isobutylamine, O-methoxyphenylethylamine, choline, and acetylcholine), purine derivatives (adenosine, adenine, guanine, caffeic acid, and amygdalin), triterpene acids, and ursolic acid [22]. Among these chemical bioactive molecules, flavonoids and procyanidins are recognized to be the key responsible components for most of the observed therapeutic effects [23, 24].

### 1.3. Toxicology

Crataegus has low toxicity, with an LD50 of 25 mg/kg [25]. Administration of excessive dosing of hawthorn flower extract (600 mg/kg/day; flavonoids) over 30 days in rats showed unremarkable adverse effects. In humans, the acute oral toxicity of hawthorn was 6 g/kg [26].

## 2. Biological Activities

It is well known that *Crataegus* species (hawthorn) have been used traditionally as a drug or supplement to promote antioxidant [27], anti-inflammatory [28], antimicrobial [29], hypoglycemic [24, 30], hypolipidemic [24], and hepatoprotective effects [31]. Treatment of atherosclerosis, urinary retention, hypertension, intestinal disorders, and brain and heart diseases have also been attributed to hawthorn [17, 18]. Hawthorn also has shown promise in the treatment of mild-to-moderate heart failure [32]. Nowadays, hawthorn is frequently used as an antidiabetic therapeutic agent in various metabolic disorders [30, 33]. The major findings suggest that the protective effects of *Crataegus* extract against diabetic-related complications most likely involve blood glucose level lowering, hypolipidemic effect, and antioxidant activity [34]and its ability to normalize insulin secretion [30, 35].

Fruit extract of hawthorn has been reported to possess lipid lowering effect. Other study showed that hawthorn leaf extract has hypolipidemic effect in diabetic models [36]. In addition, hawthorn leaf extract has been shown to have valuable hypoglycemic effect in streptozotocin- (STZ-) induced diabetic model [37] and antioxidant properties of hawthorn has been considered responsible for this beneficial effect [30]. Altogether, the underlying mechanisms of these pharmacological agents may be related to intestinal *α*-glycosidase inhibition [34, 38], decreased hepatic gluconeogenesis [39, 40], improving the lipid metabolism [39, 41], and insulin sensitivity restoration [35]. Clinically, hawthorn may be one of the future optimizing medicinal plants that shows dual effect against hyperglycemia and hyperlipidemia among human diabetic type 2 patients [42]. Data of this review have been collected from the scientific articles published in databases such as Science Direct, Scopus, PubMed, Web of Science, and Scientific Information Database from 2000 to 2021. The aim of this study is basic mechanistic information about diabetes mellitus and its complications as well as the ways which hawthorn extracts can modulate the adverse effects of diabetes.

### 2.1. Effect of Hawthorn Extracts on Diabetic-Induced Pancreatic Injury

DM is a prolonged disturbance related to irreversible destruction of islet *β*-cells insulin production, which is determined by hyperglycemia and altered fat metabolism [43]. *β*-cells in the islets of Langerhans are major location of synthesis and secretion of insulin [44]. In most individuals, a normal blood glucose concentration or normoglycemia is preserved by an increasing in insulin secretion. However, in persons predisposing to develop T2DM [45], *β*-cells unable to compensate of insulin resistance, which leads to glucose intolerance, fasting blood sugar increase, and eventually overt diabetes [46].

Three main transcription factors, including pancreas/duodenum homeobox protein 1 (PDX1), neurogenin 3 (Neurog 3), and v-maf musculoaponeurotic fibrosarcoma oncogene homolog A (MAFA), participated in early pancreatic progenitor formation, endocrine lineage specification and differentiation, and maturation of *β*-cells in the late stage, respectively [47, 48]. They contributed to reprogram of multiple cell types into insulin-producing cells for treatment of T1DM [49]. In this regard, it has been demonstrated that overexpression of PDX1, Neurog 3, and/or MAFA can induce insulin biosynthesis in various non-*β*-cells [50].

PDX1 is also essential for the differentiation of pancreatic lineages and maintenance of mature *β*-cells function [51] and also preserves pancreas against apoptosis [52]. Neurog 3 belongs to the basic helix-loop-helix transcription factor and participates in the nervous system and embryonic pancreas progress [49]. MAFA protein is a subgroup of MAF transcription family that specifically binds to insulin enhancer element RIPE3b and stimulates insulin gene expression [53].

Neurog 3 and MAFA are responsible for islet improvement. Insulin 1(Ins^−1^) and insulin 2 (Ins^−2^) promote the synthesis of proinsulin [54]. The upregulation of Ins^−1^ or Ins^−2^ may be triggered by the increased expression levels of MAFA, PDX1, and Neurog 3. It has been shown that the protein expression of MAFA, PDX1, and Neurog 3 significantly attenuated in the diabetic pancreatic tissue and treatment with *Crataegus* flavonoids (CF) at the dose of 200 mg/kg/orally for four weeks increased the protein expression pattern of MAFA, PDX1, and Neurog 3. Therefore, CF may restore the islet cells function by regulating the expression of the previously mentioned factors. CF also can ameliorate glucose intolerance and decline diet and water intake in the diabetic mice [55].

It has been observed that STZ administration leads to the islet *β*-cells toxicity and a significant reduction in the size of islets of Langerhans. The partly protective effects of CF on the islet *β*-cells are attributed to the improvement of histopathological changes of the mentioned cells [55]. Furthermore, STZ-induced diabetes is associated with deterioration of the acinar cells of the pancreas, insult to lobules, and edema. Treatment with hydroalcoholic extract of *Crataegus monogyna* at the different doses of 100, 200, and 400 mg/kg intraperitoneally for the duration of three weeks alleviated the previously mentioned histological alterations in diabetic rats [55].

### 2.2. Effect of Hawthorn Extracts on Diabetic-Induced Hepatic Injury

The liver is responsible for about 80% of endogenous glucose synthesis [56] and plays an important function in regulating of glucose homeostasis via two major mechanisms including glycogenolysis and gluconeogenesis [57]. The binding of insulin to its receptor phosphorylates PI3K/AKT pathway then regulates glycogen synthesis through inhibiting of glycogen synthase as a major enzyme in glycogenolysis. Furthermore, phosphorylation of PI3K/AKT via forkhead box protein O1 (FOXO1) inhibits phosphoenolpyruvate carboxykinase (PEPCK) and glucose 6-phosphatase (G6Pase), as key rate limiting enzymes in gluconeogenesis [58].

It has been shown, in T2DM, that hepatic insulin receptors 1A (IR-1A) deficiency is associated with an increase in gluconeogenesis, suppressing of glycogen synthesis, elevating the levels of hemoglobin A1c (HbA1_C_), and upregulating of glucose transporter 2 (GLUT2) and G6Pase mRNAs and downregulating of glycerol kinase (GK) mRNA. In addition, the activities of the glutathione (GSH) and superoxide dismutase (SOD) were decreased. Furthermore, the levels of inflammatory cytokines including tumor necrosis factor alpha (TNF-*α*) and interleukin 6 (IL-6) and thiobarbituric acid reactive substances (TBARS) in hepatic tissue were increased. The serum levels of triglyceride (TG), total cholesterol (TC), low density lipoprotein (LDL), and very low density lipoprotein (VLDL) were increased in T2DM. As indicated in Table 1, *Crataegus aronia* application at the dose of 500 mg/kg/orally for 60 days significantly reversed all aforementioned alterations to near normal, evidently through hawthorn-mediated antioxidant and anti-inflammatory actions in diabetic rats [59].

On the other hand, the hypoglycemic and hypolipidemic actions of hawthorn administration at different doses of 0.2, 0.5, and 1.0 g/kg for four weeks by oral administration regulated the expression of hepatic gluconeogenesis and lipogenesis associated genes including sterol regulatory element binding protein-1c (SREBP-1c) and fatty acid synthase (FAS). Furthermore, blood glucose lowering effect of hawthorn may be attributed to attenuating of gluconeogenesis in the liver tissue through downregulating of PEPCK gene expression following AMP-activated protein kinase (AMPK) activation [39].

Peroxisome proliferator-activated receptor *α* (PPAR*α*), as a nuclear receptor, has a pivotal role in lipid metabolism [60], especially in hepatic lipid metabolism through the induction of various genes such as fatty acid transport proteins and fatty acid oxidation cascades [61]. PPAR*α* also has an essential function in SREBP-mediated regulation of lipogenic genes [62]. Furthermore, PPAR*α* reduces the serum levels of TG, while it elevates high-density lipoprotein-cholesterol (HDL-C) in patients with dyslipidemia [63]. In this regard, it has been shown that hawthorn effectively increases the mRNA expression levels of PPAR*α* in the liver tissue of high fat diet mice model, whereas it inhibits the expression of SREBP1c and SREBP2 contributed to the triacylglycerol and total cholesterol synthesis, respectively [39].

### 2.3. Effect of Hawthorn Extracts on Diabetic-Induced Gastric Injury

Gastroparesis is explained as a syndrome specified by dysfunction in stomach resulting in delayed gastric emptying without mechanical obstruction [64]. Diabetic gastroparesis (DGP) is a well-established chronic side effects of diabetes. It has been reported that 30–50% of patients with T1DM or T2DM suffer from gastroparesis [65]. Furthermore, the risk of developing DGP, being over 7 and 30-times for T2DM and T1DM, respectively. DGP is a highly symptomatic disorder determined by fullness, anorexia [66], early satiety, abdominal pain [67], weight loss, abdominal distension, nausea and vomiting [68], gastric dysrhythmia, antral hypomotility, and/or delayed gastric emptying [65].

Numerous factors such as autonomic system neuropathy, disturbance in neurotransmission [69], hyperglycemia, neurological disorders, alterations in gastrointestinal (GI) hormones, microvascular and GI smooth muscle abrasions, and structural and practical irregularity of interstitial cells of Cajal (ICC) are involved in the pathogenesis of DGP [68]. Hyperglycemia-induced oxidative stress is a major mechanism for various complications of diabetes including DGP and leads to cellular malfunction and tissue damage [70]. The role of three main antioxidant enzymes such as SOD, catalase (CAT), and glutathione peroxidase (GP_X_), as the first line defense systems, are important in animals [71]. The beneficial effect of these antioxidants on GI disorder was reported [72] and may be also effective for DGP [70].

**Figure 1:**
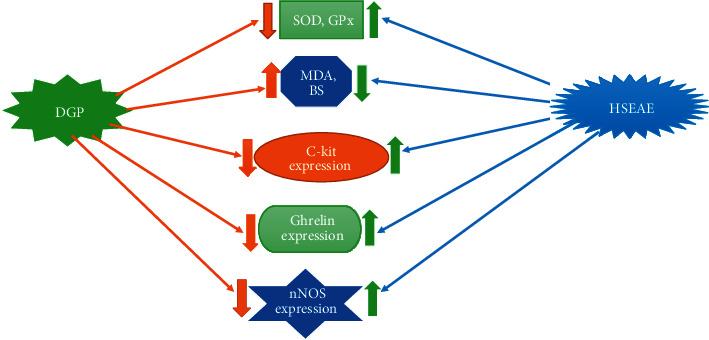
Schematic presentation of HSEAE on DGP and possible mechanisms.

It is revealed that the antioxidant activity of SOD and GP_X_ decreased and the level of malondialdehyde (MDA), as a major indicator of stress oxidative, enhanced significantly in underlying of DGP. As shown in Figure 1, the oral administration of ethyl acetate extract hawthorn seeds (HSEAE) at the doses of 3, 6, and12 mg/kg for four weeks reverses all these changes in diabetic rats. This implies that administration of HSEAE can beneficially suppress oxidative stress [68].

Several hormones secreted by the GI system participate in regulating of GI motor activity [73]. Ghrelin is an endogenous ligand of growth hormone releasing hormone receptor, released into the bloodstream from the stomach. Ghrelin activates growth hormone and gastric acid secretion via the hypothalamus and accelerates GI peristalsis and food intake [74]. It is presented that plasma and gastric ghrelin expression in DGP significantly were downregulated but dramatically upregulated following administration of HSEAE [68].

Earlier study showed that reduction of neuronal nitric oxide synthase (nNOS) activity or protein expression in the gastric neurons of DGP results in a decrease in the nitric oxide (NO) production, which decelerates gastric activity [75]. In this regard, one experimental study showed that the administration of extract of hawthorn seeds increased the decreased protein expression of nNOS in the gastric of DGP subject, which may be advantage for facilitating gastric motility [68]. ICCs are the pacemaker for gut motility. They have beneficial effects on contractile activity of stomach muscle and eventually promote the gastric emptying [76]. ICCs are identified by mature molecular marker c-kit. Downregulation of c-kit expression critically influences the normal function, growth, and maturity of ICCs and declines their number and disturbances ultrastructural [66]. It has been revealed that the expression of c-kit protein in the DGP subjects was significantly downregulated and upregulated after theadministration of HSEAE [68].

The molecular mechanism underlying ethyl acetate extract hawthorn seeds (HSEAE, 3, 6, and12 mg/kg/oral/4 weeks/rat) on diabetic gastroparesis (DGP) may be related to elevating the antioxidant enzymes activity of superoxide dismutase (SOD), catalase (CAT), upregulating expression of C-kit, ghrelin, nitric oxide synthase (nNOS), and also attenuating the blood glucose (BS) and malondialdehyde (MDA) levels.

### 2.4. Effect of Hawthorn Extracts on Diabetic-Induced Cardiac Injury

Diabetic cardiomyopathy (DCM) is determined by a direct damage to cardiac muscle and subsequently structural and practical alterations in myocardium in absence of hypertension and other cardiovascular problems. Hyperglycemia has a pivotal role in the pathogenesis of DCM. Cardiac hypertrophy, ventricular electrophysiological disturbances, and heart failure are the most important complications of DCM [77].

It has been reported that DCM is accompanying with oxidative stress, expression levels of inflammatory proteins, apoptosis, and accumulation of extracellular matrix [78]. Hyperglycemia results in reactive oxygen species (ROS) overproduction that led to the oxidative stress-related cardiac injury and major changes in myocardial cells, and consequently DCM. Antioxidants can eliminate a little amount of ROS in the normal physiological situation, but in pathological conditions for example in diabetes, because of the excessive ROS production, antioxidants cannot remove ROS and therefore induce insult to heart cells [79]. It is revealed that the activity of SOD, one of the most significant antioxidant enzymes [80], markedly decreased following DCM. Pretreatment with doses of 50, 100, and 200 mg/kg of hawthorn leaf flavonoids (HLF) for 16 weeks significantly increased the activity of SOD and decreased the increased levels of MDA in underlying DCM [81].

Protein kinase C (PKC) plays a fundamental role in regulation of multiple cellular processes [82]. Evidence indicated that the PKC activation can be involved in the cardiovascular injury through increasing the synthesis of extracellular matrix, regulating the calcium ion metabolism of cardiac cells, stimulating angiotensin II, and inducing ROS generation and inflammatory agents [83, 84]. The mRNA and protein expression of PKC-*α* were evaluated following experimental DCM. It is observed that the mRNA and protein expression of PKC-*α* in the cytoplasm and cytomembrane of cardiac cells dramatically increased. Pretreatment with HLF significantly downregulates these parameters in underlying DCM [81].

Inflammation participates in the progress of many cardiovascular disorders including hypertension, atherosclerosis, ischemic heart diseases, and congestive heart failure [85]. Furthermore, inflammatory cytokines have a key role in cardiac disturbance following myocardial infarction [86]. The correlation between TNF-*α* and nuclear factor kappa B (NF-*κ*B) has been reported in myocardial dysfunction in underlying diabetes. The stimulated NF-*κ*B can dramatically upregulate the expression of TNF-*α* in left ventricular myocardium of diabetic subjects; however, TNF-*α* activates overexpression of NF-*κ*B [87]. Experimental evidence illustrated that HLF treatment significantly downregulates the overexpression of NF-*κ*Bp65 and TNF-*α* in cardiac tissue after induction of diabetes [81].

The cardioprotective actions of *Crataegus oxyacantha* extract on cardiac ischemia/reperfusion (I/R) damage in underlying diabetes has been investigated. The advantage effects may be related partly to antioxidant effects of hawthorn [88]. Lactate dehydrogenase (LDH) and creatine kinase myocardial band isoenzyme (CK-MB) are the most sensitive and specific indices for the evaluation of cardiac insult. Myocardial cells death leads to a pivotal increase in the serum levels of these enzymes [89]. Scientific evidence showed that the serum levels of LDH and CK-MB significantly increased after cardiac I/R injury associated with diabetic induction. Moreover, this study suggested that the previously mentioned indicators dramatically reduce following the oral administration of *Crataegus oxyacantha* extract at the dose of 100 mg/kg for 10 weeks in diabetic rats [88].

Oxidative stress contributed in I/R injury development, and these effects aggravated in diabetes [90]. Myeloperoxidase (MPO) as an oxidative stress parameter leads to ROS generation. Combination treatment of *Crataegus oxyacantha* extract with resistance training significantly attenuated the increased levels of MPO following cardiac I/R insult in underlying diabetes [88]. Experimental study indicated that diabetes induced-oxidative stress reduces the activity of GPx, which leads to the aggregation of hydrogen peroxide levels [91]. GPxas, a major ROS scavenger, accelerates the oxidation of glutathione via cumene hydroperoxide. It is reported that *Crataegus oxyacantha* extract, along with resistance training, significantly increased the decreased activity of GPx [88].

### 2.5. Effect of Hawthorn Extracts on Diabetic-Induced Vascular Injury

**Figure 2:**
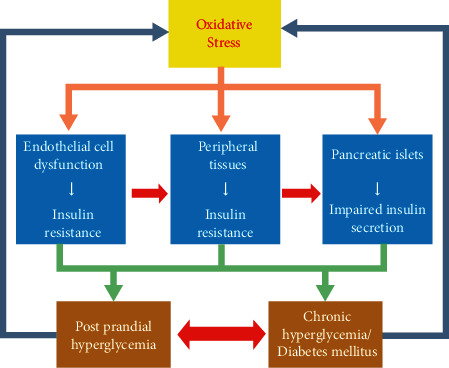
Schematic presentation of correlation between oxidative stress and DM. Oxidative stress displays dangerous effects on *β*-cells of pancreatic islets, adipocytes, and peripheral tissues.

Insulin resistance (IR) is a major etiology and pathogenesis for T2DM. Development of IR is mostly associated with oxidative stress and/or inflammatory reactions induced by numerous proinflammatory agents such as interleukin-1 beta (IL-1*β*), IL-6, and TNF-*α* [92]. Oxidative stress results in a mismatch between the ROS generation and antioxidant system. Pancreatic *β*-cells, adipocytes, and peripheral tissues are more susceptible to the destruction effects of oxidative stress [93]. Oxidative stress leads to impairment of insulin secretion in *β*-cells and IR progress in adipocytes and peripheral tissues that eventually accelerates postprandial hyperglycemia and overt T2DM. As shown in Figure 2, both postprandial hyperglycemia and T2DM act as feedback loop for the occurrence of oxidative stress [92]. It has been indicated the potential therapeutic or preventive effects of polyphenols as free radical scavengers in diabetes and its complications [94].

These events are associated with impairment of insulin secretion in *β*-cells and development of insulin resistance in adipocytes and peripheral tissues, which lead to the progress of postprandial hyperglycemia and overt T2DM, both of which also operate as feedback loop for the occurrence of oxidative stress [92].

It has been shown that hawthorn polyphenols extract (HPE) at the dose of 300 mg/kg for four weeks can decrease the progression of T2DM by attenuating ROS and increasing the CAT, GP_X_, and total antioxidant capacity (TAC) in serum and colonic tissue of diabetic rats model. This study also confirmed that HPE can effectively regulate altering parameters such as fasting blood sugar (FBS), oral glucose tolerance test (OGTT), TG, TC, insulin, lipopolysaccharide (LPS) serum levels, and body weight [95]. GLUTs facilitate absorption of glucose from the circulation into the cells. GLUT4 is essentially expressed in skeletal muscles, adipocytes, and cardiomyocytes. GLUT4 particularly stimulates glucose uptake into the muscle and adipose cells [96].

Insulin receptor substrate (IRS) family has six proteins (IRS1-IRS6). IRS1 and IRS2 are essential in insulin signaling pathways [97]. IRS-1 plays a pivotal role in activation of phosphatidylinositol 3-kinase (PI3K) as the active center of most insulin metabolic actions [98]. It is well established that attachment of insulin-to-insulin receptors is associated with tyrosine phosphorylation of IRS-1, which advances signal transduction via the IRS/PI3K signaling pathway and leads to glucose and fat metabolism [99]. Furthermore, stimulation of the PI3K/AKT pathway increases insulin secretion from pancreatic *β*-cells [100]. In this regard, it has been shown that PI3K can enhance AKT kinase (protein kinase B or PKB) activity by stimulating AKT tyrosine phosphorylation and finally provoke glucose transport, lipogenesis, glycogen synthesis, and repression of gluconeogenesis [98].

On the other hand, it is indicated that disturbance of translocation of GLUT4, as a downstream target of IRS1/PI3K/AKT signaling pathway, from intracellular to cytomembrane is one of the important etiology of IR in T2DM [101]. Another study presented that IR can improve through the stimulation of IRS1-PI3K-AKT-GLUT4 network in diabetic model [102]. In addition, it has been observed that HPE effectively enhances the phosphorylation of GLUT4 and IR-associated proteins such asIRS1, AKT, and PI3K in the liver tissue and dramatically increases the expression levels of phosphorylation (p)-IRS1 and p-AKT in the skeletal muscle of diabetic rats [95].

Besides, it has been described that inflammatory markers, including IL-6, TNF-*α*, and monocyte chemoattractant protein-1 (MCP-1), contributed to IR. These proinflammatory cytokines interfere with insulin signaling networks and influence the insulin signaling kinase action [103]. In addition, chronic inflammation in adipose, liver, and skeletal muscle tissues are involved in the incidence and pathogenesis of T2DM [104]. It is investigated that under action of HPE the serum levels of IL-6 and TNF-*α* as well as protein expression of TNF-*α* and IL-6 in skeletal muscle and liver significantly mitigate in the diabetic model. This polyphenol also attenuates the protein expression of MCP-1 in the hepatocytes [95].

NF-*κ*B is one of the major transcriptional mediated pathways, which participates in numerous inflammatory reactions [105]. It accelerates the expression of downstream target proinflammatory cytokines, such as TNF-*α*, IL-6, and MCP-1 in the nucleus [95].

The activation of NF-*κ*B signaling cascade in the aorta of diabetic model has been investigated and reported that phosphorylation of I*κ*B, as inhibitor of NF-*κ*B, and breakdown of its lead to NF-*κ*Bp65 entrance to the nucleus, which induces vascular injury and upregulates proinflammatory markers expression [106]. HPE through decreasing the protein expression of NF-*κ*Bp65 phosphorylation attenuates the expression of aortic inflammatory agents in the diabetic model [95]. On the other hand, it has been investigated that sirtuin 1 (SIRT1)/AMPK pathway decreases the transcriptional stimulation of NF-*κ*B [107]. It has been reported that activation of AMPK regulates glucose homeostasis through inhibition of gluconeogenesis and attenuation of glucose levels [108].

**Figure 3:**
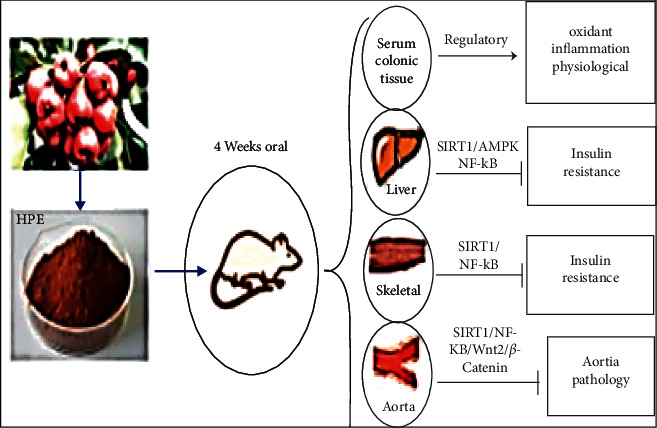
Molecular mechanisms of HPE on diabetic-aorta injury [95].

SIRT1 is one of most members of sirtuin (SIRT1-7) family whose enzymatic activity is related to nicotinamide adenosine dinucleotide (NAD+) cofactor. It is indicated that upregulation of SIRT1improves insulin sensitivity [109]. Moreover, interaction of SIRT1with p65 subunit of NF-*κ*B and its deacetylation inhibits NF-*κ*B downstream signaling pathway [110]. As shown in Figure 3, HPE attenuates chronic inflammation in the liver and skeletal muscle via the SIRT1/AMPK/NF-*κ*B and SIRT1/NF-*κ*B signaling pathways, respectively, thereby suppressing IR in the diabetic model [95].

Diabetic-induced vascular insult is one of the etiologies of mortality rate among patients with diabetes [111]. Wnt/*β*-catenin signaling cascades participate in macrovascular injury [112]. The interaction of Wnt ligand with the transmembrane receptors stabilises cytoplasmic *β*-catenin and ultimately initiates the transcription of downstream target genes of Wnt pathway [113]. It is reported that aberrations of Wnt/*β*-catenin signaling pathways are associated with glucose metabolism disorders and increase the risk of T2DM [114]. On the other hand, Wnt/*β*-catenin signaling pathway is stimulated by upregulation of SIRT1 [115]. It is observed that HPE contributed to aortic damage repair via modulating the NF-*κ*B, downregulating the Wnt/*β*-catenin signaling pathway, regulating the expression of its downstream associated protein *ks*, and stimulating the regulatory action together with SIRT1 in diabetic model [95].

Pretreatment with hawthorn polyphenol extract (HPE) via oral administration for four weeks has regulatory effects on oxidant and inflammation agents in serum and colonic tissue and suppresses insulin resistance through inhibition of SIRT1/AMP-activated protein kinase (AMPK)/nuclear factor kappa B (NF-*κ*B) pathway in the liver and SIRT1/NF-*κ*B cascade in the skeletal muscles of diabetic rats model. HPE also repairs aortic injury via SIRT1/NF-*κ*B/Wnt2/*β*-catenin pathway.

### 2.6. Effect of Hawthorn Extracts on Diabetic-Induced Renal Injury

The kidney has a substantial role in glucose handling through gluconeogenesis, glucose filtration, glucose reabsorption, and glucose utilization. Each of these ways can be changed in patients with T2DM [117]. As mentioned earlier, DM is determined by hyperglycemia, polyuria (an excessive urination), polydipsia (a great thirst), polyphagia (an enhanced appetite), and glycosuria [118]. Diabetic kidney disease (DKD) is a danger kidney-related complication of DM, which leads to end-stage renal disease [119]. Multiple factors, such as oxidative stress [120], genetic factors, glucose metabolism disorder, alteration in hemodynamic, and inflammation, participate in the pathogenesis of DKD [121].

Diabetic-induced oxidative stress leads to accumulation of ROS in kidney cells and subsequent stimulated p38 mitogen activated protein kinase (p38MAPK), which is involved in cellular stress, inflammation, and apoptosis through various processes and further exacerbates DKD progression [122]. On the other hand, it has been reported that, following the diabetic nephropathy, renal cell activity of p38 quickly increases in glomeruli, tubules, and renal interstitial cells [123]. Previous study showed that suppression of the p38MAPK signaling pathway can dramatically attenuate the risk of proteinuria in DKD, decrease the secretion of inflammatory mediators, and eventually decline DKD development [124].

**Figure 4:**
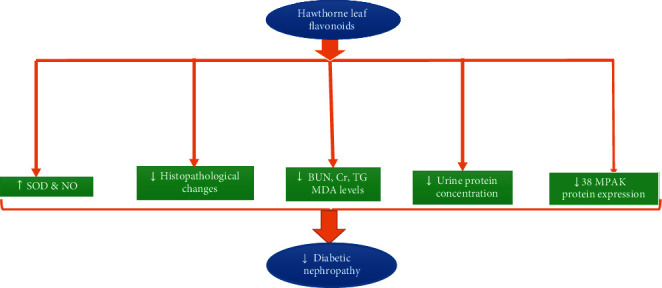
Effect of hawthorn extracts (200 mg/kg/oral/12 weeks) on the diabetic nephropathy.

Previous literature pointed out that HLF, at the dose of 200 mg/kg for 12 weeks through oral administration, can increase antioxidant capacity and prevent the process of lipid peroxidation in diabetic rats [116]. Another study showed that pretreatment with HLF effectively decreased the increased p38MAPK protein expression in the renal tissue of DKD. As shown in Figure 4, HLF also significantly decreased the elevated serum biomarkers, including blood urea nitrogen (BUN), creatinine (Cr), TG, MDA levels, and urine protein in DKD. Moreover, HLF effectively increased the reduced SOD, NO levels, body weight, and improved histopathological changes of DKD [116].

The possible mechanisms of hawthorn leaf extracts on diabetic nephropathy attributed to increasing SOD and nitric oxide (NO, and decreasing the blood urea nitrogen (BUN), creatinine (Cr), triglyceride (TG), MDA levels, urine protein concentration, 38mitogen activated protein kinase (38MPAK) protein expression, and improving the histopathological changes of kidneys tissue.

### 2.7. Effect of Hawthorn Extracts on Diabetic-Induced Cerebral Injury

DM is considered as a predisposing factor for cognitive disturbance [125] and the progress of Alzheimer's disease and dementia [126]. Furthermore, passive avoidance learning (PAL) and memory defective take place in STZ-induced diabetes [127]. The analgesic effects of hawthorn extract on spontaneous locomotors activities and exploratory behaviors have been investigated [128]. In this relation, it is reported that *Crataegus* extract at the doses of 100, 300, and 1000 mg/kg for two weeks by oral administration ameliorated PAL and attenuated time spent in the dark compartment of diabetic rat model [24]. Furthermore, hawthorn extract and resistance training improved cognitive deficits in STZ -induced diabetic rats [129].

It has been demonstrated that hawthorn extract supplementation decreased the increased BS, obesity-related factors, including TG and cholesterol and increased the decreased density HDL levels in the serum of diabetic model [24]. The effects of hawthorn extract on lipid metabolism may be attributed to the modulating of lipoprotein lipase (LPL) expression through peroxisome proliferator response element cascade [130] and suppressing synergistically 3-hydroxy-3-methylglutaryl-coenzyme A (HMG-CoA) reductase and cholesterol assimilation [131]. Another study showed that flavonoids are one of the major components of hawthorn extracts, which operate as an alpha-amylase inhibitor, glycemic management and have beneficial effects on dyslipidemia in diabetic model [132].

## 3. Conclusion

Based on multiple reports as mentioned in this manuscript, hawthorn extracts supplementation significantly improved diabetic-induced injuries in several organs, such as pancreases, stomach, liver, heart, vessel, kidneys, and brain. The findings suggest that the effects of *Crataegus* extracts against diabetic-related complications are most likely related to improving the physiological functions, including enhancement of body weight, insulin secretion, OGTT, blood glucose level lowering, hypolipidemic action via decreasing TG, cholesterol, and antioxidant effect through increasing the activity of SOD, CAT, GP_X_, and TAC and decreasing MDA level and anti-inflammatory effects through attenuating TNF-*α*, IL-6, and SIRT1/AMPK/NF-*κ*B pathway. Other mechanisms are increasing the phosphorylation of GLUT4, IRS1, AKT, and PI3K and attenuating the urine protein concentration, p38MPAK protein expression, BUN, and Cr and improving the histopathological changes. Collectively, hawthorn extracts can be regarded as one new target for the research and development of innovative drugs to the prevention or treatment of DM and related complications.

## Figures and Tables

**Table 1:** Effects of hawthorn extracts on diabetic-induced injury in several organs.

Organ	Hawthorn extract	Dose(s)/route/duration/animal	Effects	Reference (s)
Stomach	Ethyl acetate extract hawthorn seeds	3, 6, and12 mg/kg/oral/4 weeks/rat	↓BS&MDA SOD and GP_X_↑ ↑ plasma level and ghrelin gastric expression ↑ nNOS gastric expression c-kit gastric expression ↑ Gastric emptying and small intestinal ↑ propulsion ↑ Body weight	[68]

Liver	*Crataegus aronia*	500 mg/kg/60 days/oral/rat	↓ BS,HbA1_C_, OGTT, TG, TC,LDL, VLDL, TBARS, TNF-*α*&IL-6 ↑GSH, SOD, HDL, and hepatic glycogen ↓GLUT-2 and G6Pase hepatic mRNA_S_ ↑IR-A and GK hepatic mRNA_S_	[59]

Liver	*Crataegus pinnatifida Bge. var. major N.E. Br.*	0.2, 0.5, 1.0 g/kg/4 weeks/oral/rat	↑ AMPK phosphorylation& HDL-C PPARs expression↑ ↓ PEPCK and glucose production ↓ SREBP-1c, SREBP2, and FAS	[39]

Pancease	*Crataegus monogyna* extract	100, 200, and 400 mg/kg/ip/3 weeks/rat	↓ BS and MDA TAC↑ ↓ histopathological alterations	[55]

Pancease	*Crataegus* flavonoids	200 mg/kg/oral/4 weeks/mice	↑ Serum insulin levels ↑ PDX-1, Mafa and Neurog 3, Ins-1, and Ins-2 ↓Appetite ↓ Water intake ↓ OGTT ↓ Histopathological alterations	[55]

Heart	Hawthorn leaf flavonoids	50, 100, and 200 mg/kg/oral/16 weeks/rat	↓ BS and MDA ↓TNF-*α* and NF-*κ*B proteins expression ↓ mRNA expression of PKC-*α* & PKC-*α* protein ↓ Histopathological changes SOD ↑	[81]

Heart	*Crataegus oxyacantha* extract	100 mg/kg/oral/10 weeks/rat	↑ Body weight ↑ GPx ↓ FBS, CK-MB, and LDH ↓ MPO	[88]

Aorta	Hawthorn polyphenol extract	300 mg/kg/oral/4 weeks/rat	↓ TC, TG, FBS, OGTT, and LPS levels ↑ Insulin level and body weigh ↑ Phosphorylation of GLUT4, IRS1, AKT, and PI3K in liver ↓ TNF-*α* and IL-6 in serum ↑ Phosphorylation of IRS1 and AKT in skeletal muscle ↓ Protein expression of TNF-*α* and IL-6 in liver and skeletal muscle ↓ Protein expression of MCP-1 in the liver Repair of aortic injury via SIRT1/NF-*κ*B/Wnt2/*β*-catenin pathway ↓ Inflammation and IR (liver): SIRT1/AMPK/NF-*κ*B pathway ↓ Inflammation &IR(skeletal muscle): SIRT1/NF-*κ*B pathway	[95]

Kidney	Hawthorne leaf flavonoids	200 mg/kg/oral/12 weeks/rat	↑ Body weight ↑ SOD and NO ↓ Urine protein concentration ↓ BUN, Cr, TG& MDA levels ↓ p38MPAK protein expression ↓Histopathological changes	[116]

Brain	*Crataegus* extract	100, 300, and 1000 mg/kg/oral/2 weeks/rat	↓Cholesterol, TG, and BS ↑ HDL Improvement of PAL	[24]

## Abbreviations

DM: Diabetes mellitus
T1DM: Type 1 diabetes mellitus
T2DM: Type 2 diabetes mellitus
PDX1: Pancreas/duodenum homeobox protein 1
Neurog 3: Neurogenin 3
Ins^−1^: Insulin ^1^
Ins^−2^: insulin ^2^
CF: Crataegus flavonoids
STZ: Streptozotocin
FOXO1: Forkhead box protein O1
PEPCK: Phosphoenolpyruvate carboxykinase
G6Pase: Glucose 6-phosphatase
IR-1A: Insulin receptors 1A
HbA1_C_: Hemoglobin A1c
GLUT2: Glucose transporter 2
GK: Glycerol kinase
GSH: Glutathione
SOD: Superoxide dismutase
TNF-*α*: Tumor necrosis factor alpha
IL-6: Interleukin 6
TBARS: Thiobarbituric acid reactive substances
TG: Triglyceride
TC: Total cholesterol
LDL: Low density lipoprotein
VLDL: Very low-density lipoprotein
SREBP-1c: Sterol regulatory element binding protein-1c
FAS: Fatty acid synthase
AMPK: AMP-activated protein kinase
PPAR*α*: Peroxisome proliferator-activated receptor *α*
HDL-C: High-density lipoprotein-cholesterol
DGP: Diabetic gastroparesis
GI: Gastrointestinal
ICC: Interstitial cells of Cajal
CAT: Catalase
GPX: Glutathione peroxidase
MDA: Malondialdehyde
HSEAE: Ethyl acetate extract hawthorn seeds
NNOS: Neuronal nitric oxide synthase
NO: Nitric oxide
DCM: Diabetic cardiomyopathy
ROS: Reactive oxygen species
HLF: Hawthorn leaf flavonoids
PKC: Protein kinase C
NF-*κ*B: Nuclear factor kappa B
I/R: Ischemia/reperfusion
LDH: Lactate dehydrogenase
CK-MB: Creatine kinase myocardial band isoenzyme
MPO: Myeloperoxidase
IR: Insulin resistance
IL-1*β*: Interleukin-1 beta
HPE: Hawthorn polyphenols extract
TAC: Total antioxidant capacity
FBS: Fasting blood sugar
OGTT: Oral glucose tolerance test
LPS: Lipopolysaccharide
IRS: Insulin receptor substrate
PKB: Protein kinase B
MCP-1: Monocyte chemoattractant protein-1
SIRT1: Sirtuin 1
NAD+: Nicotinamide adenosine dinucleotide
DKD: Diabetic kidney disease
p38MAPK: p38 mitogen activated protein kinase
BUN: Blood urea nitrogen
Cr: Creatinine
PAL: Passive avoidance learning
LPL: Lipoprotein lipase
HMG-CoA: 3-hydroxy-3-methylglutaryl-coenzyme A.
